# Giant Cell Fibroma of Tongue: Understanding the Nature of an Unusual Histopathological Entity

**DOI:** 10.1155/2014/864512

**Published:** 2014-01-09

**Authors:** Wanjari Ghate Sonalika, Anshuta Sahu, Suryakant C. Deogade, Pushkar Gupta, Dinesh Naitam, Harsh Chansoria, Jatin Agarwal, Shiva Katoch

**Affiliations:** ^1^Department of Oral Pathology and Microbiology, Hitkarini Dental College and Hospital, Jabalpur, Madhya Pradesh 482001, India; ^2^Department of Oral Pathology and Microbiology, Maitri College of Dentistry and Research Center, Durg, Chattisgarh 490020, India; ^3^Department of Prosthodontics, Hitkarini Dental College and Hospital, Jabalpur, Madhya Pradesh 482001, India; ^4^Department of Prosthodontics, Rungta College of Dental Sciences and Research, Bhilai, Chattisgarh 490024, India; ^5^Department of Prosthodontics, Government College of Dentistry, Indore, Madhya Pradesh 452001, India; ^6^Department of Prosthodontics, Shri Aurbindo Institute of Medical Sciences, Indore, Madhya Pradesh 453555, India; ^7^Department of Prosthodontics, M.N.D.A.V Dental College and Hospital, Solan, Himachal Pradesh 173223, India

## Abstract

Giant cell fibroma (GCF) is a rare case with unique histopathology. It belongs to the broad category of fibrous hyperplastic lesions of the oral cavity. It is often mistaken with fibroma and papilloma due to its clinical resemblance. Only its peculiar histopathological features help us to distinguish it from them. The origin of the giant cell is still controversial. Data available is very sparse to predict the exact behavior. Hence, we report a case of GCF of tongue in a 19-year-old male. Special emphasis is given to understand the basic process of development of the lesion, nature of giant cells, and also the need for formation of these peculiar cells. Briefly, the differential diagnosis for GCF is tabulated.

## 1. Background

Giant cell fibroma (GCF) is an unusual fibrous mucosal mass with several unique features separating it from other oral fibrous hyperplasias [1]. First reported by Weathers and Callihan in 1974 [2], GCF is found predominantly in Caucasians in first three decades of life with slight female predilection. The etiology for GCF remains unknown and does not appear to be associated with chronic irritation [1]. It typically manifests as an asymptomatic sessile or pedunculated mass [1] that is commonly mistaken for other growths such as fibroepithelial polyp, pyogenic granuloma, and fibroma [3] and can be diagnosed accurately based only on its distinctive histopathology.

Herewith, we report a case of GCF of tongue in a 19-year-old male, along with simultaneous comparison with irritation fibroma and retrocuspid papilla. Additionally, adding epidemiological data to the literature can help predict the exact nature of this relatively uncommon entity.

## 2. Case Presentation

**Figure 1:**
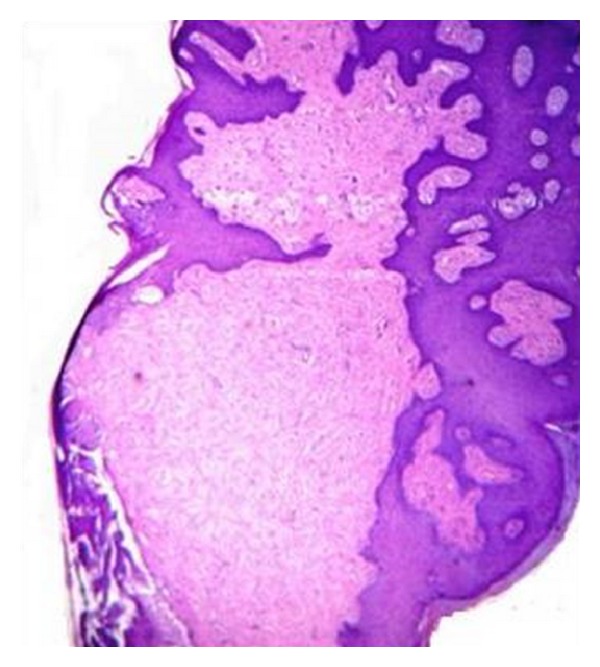
Photomicrograph showing a fibrous mass with overlying stratified squamous epithelium with elongated rete ridges. (Hematoxylin and Eosin, original magnification 4x).

**Figure 2:**
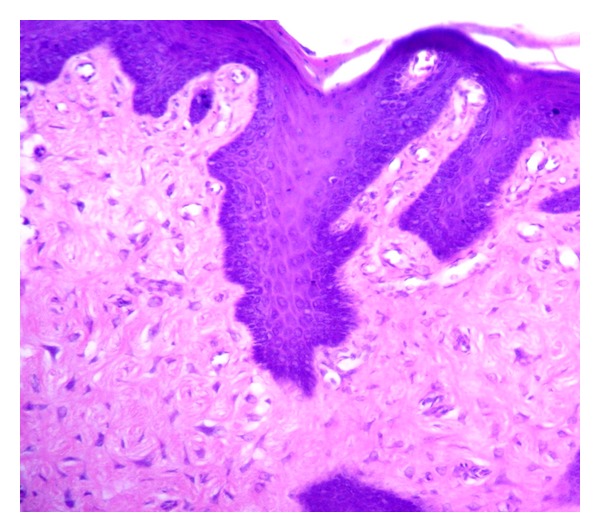
Photomicrograph showing dense collagen fibers with numerous giant cells, especially near epithelium. (Hematoxylin and Eosin, original magnification 10x).

**Figure 3:**
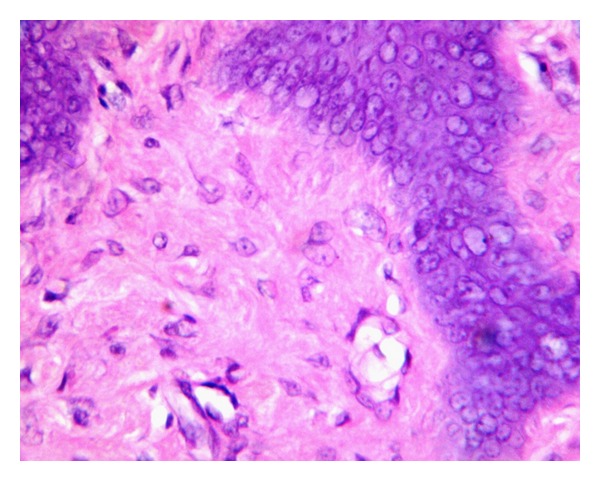
Photomicrograph showing giant fibroblasts with stellate shape and some contains two nuclei. (Hematoxylin and Eosin, original magnification 40x).

A 19-year-old male reported with a small growth on the tip of the tongue. The growth was round in shape, measuring approximately 1 mm × 0.5 mm, smooth surfaced, normal mucosal colour and sessile. It was nontender and firm in consistency with no history of trauma. A clinical diagnosis of fibroma was given and was subjected to excisional biopsy. Histopathological examination of the excised specimen revealed a relatively avascular fibrocellular connective tissue mass. The surface epithelium was hyperplastic stratified squamous with elongated and thin rete ridges (Figure 1). Characteristically, the stroma consisted of numerous giant cells especially near the surface epithelium (Figure 2). The giant cells were stellate shaped with dendritic process, containing moderate amount of basophilic cytoplasm and large vesicular nuclei with prominent nucleoli. Few giant cells were binucleated (Figure 3). Based on these features a final diagnosis of giant cell fibroma was given. The patient is under regular follow-up and no recurrence is reported after 11 months of follow-up.

## 3. Discussion

Fibrous hyperplastic lesions are encountered commonly in the oral cavity [1] and can appear similar both clinically and histologically. They comprise a diverse group of reactive and neoplastic conditions. Amongst these, irritation fibroma, a reactive lesion is the most common to occur [4] but its histopathological variant known as giant cell fibroma is a rare entity.

GCF commonly affects Caucasians; other races are rarely involved [5]. GCF shows a slight female predominance with female to male ratio of 1.2 : 1 [6] but few studies [5, 7] have reported equal sex predilection. The most common location is the gingiva with tongue being the second most common location, followed by the buccal mucosa or palate [1, 8]. Usually it manifest as an asymptomatic, sessile, or pedunculated lesion measuring about 0.5 to 1 cm with a bosselated or pebbly surface [6]. Our case had comparable findings. The exact etiology is largely unknown, but few authors have suggested trauma or chronic irritation as the inciting factors [9] whereas few authors rule out these factors [1, 8]. A possible viral origin [9] for the tumor is also postulated.

Histologically, GCF is characterized by the presence of numerous large stellate and multinucleated giant cells in a collagenous stroma of varying density. The giant cells are usually seen numerous in the connective tissue immediately adjacent to the epithelium. These giant cells have well-defined cell borders and show dendritic processes. Some of these cells, especially those located subjacent to the epithelium may contain small brown granules having staining characteristics of melanin [10]. An artifactual space separating the giant fibroblasts from the surrounding fibrous stroma is sometimes seen. The overlying epithelium is hyperplastic with thin elongated rete ridges. Inflammatory infiltrate is usually absent [1, 3].

To understand the exact nature of these giant cells various electron microscopic and immunohistochemical studies have been performed. Ultrastructural findings [11, 12] are in accordance with the light microscopic findings of stellate shaped, multinucleated giant cells with hyperchromatic nucleus, distinct cell borders, and dendritic-like cytoplasmic extension. Additionally, the cells showed numerous intracellular microfibrils thus supporting the fibroblastic nature of these cells. Immunohistochemical studies [9, 13–16] have also confirmed the fibroblastic lineage of these giant cells as evident by vimentin positivity of these cells. Earlier, when Weathers and Callihan (1974) [2] first reported GCF, they suggested that the giant cells might be melanocytes or langerhans cells. This was further supported by Houston [17] in 1982. But the negative staining for S-100 obtained by several investigators [7, 14, 15] ruled out this theory. Endothelial and myofibroblastic origin was ruled out by negative staining for alpha-smooth muscle actin. Negativity for CD68, Leukocyte common antigen (LCA) and HLA-DR [15] overrules macrophage-monocyte lineage.

Although the fibroblastic origin of these giant cells is clear, the reason as to why these giant cells are formed still remains uncertain. Fibroblast plays multifunctional and dynamic role during wound healing process and is the main cell influencing the extracellular matrix protein synthesis. Tettamanti et al. [18] described the ultrastructure of a stimulated fibroblast as stellate shaped due to the cytoplasmic membrane laminae whereas the quiescent fibroblasts were spindle shaped. Substantial evidence is present at light microscopic and electron microscopic level which indicates that the giant cells are active cells. The stellate morphology, presence of vesicular nucleus with prominent nucleoli, basophilic cytoplasm due to high mRNA content characterizes a cell which is involved actively in synthesis process. Positivity for prolyl-4-hydroxylase obtained by Odell et al. [14] further supports the finding. They concluded that these giant cells show a functional fibroblast differentiation.

**Figure 4:**
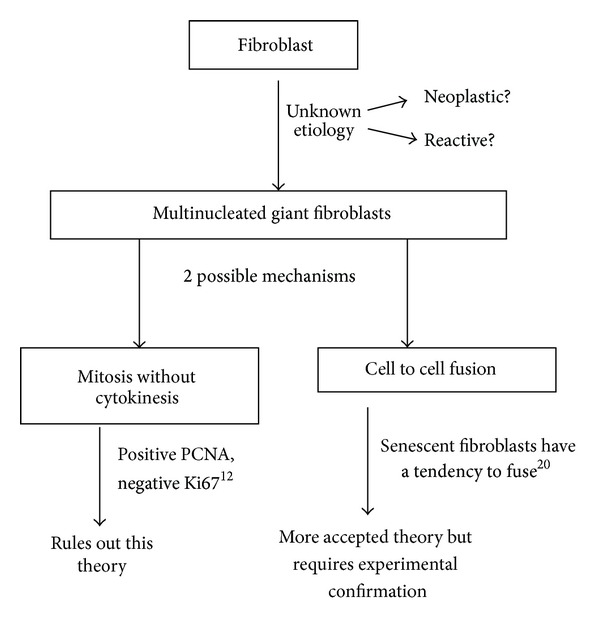
Schematic reprsentation showing possible histogenetic mechanisms for giant fibroblasts.

Histogenesis of multinucleation in the giant fibroblasts also remains unclear. There are two widely accepted mechanisms by which multinucleated cells are formed viz: cell to cell fusion and mitosis without cytokinesis. Holt and Grainger (2011) [19] have proved experimentally that in culture, fibroblasts can from multinucleated cells by both mechanisms. But the immunohistochemical analysis done by Mighell et al. [13] has shown positivity for proliferating cell nuclear antigen but Ki-67 was negative inferring that mitosis without cytokinesis is not involved in the formation of giant cells of GCF. Thus, the alternate hypothesis of multinucleated giant cell formation via cell fusion could possibly be the histogenetic mechanism in GCF cases. But further confirmation is needed for acceptance of such a hypothesis. Multinucleation is also seen in senescent fibroblasts probably resulting from damage to the mitotic machinery of these cells. Experiments have shown presence of multinucleated fibroblasts in aged periodontal ligament. Cho and Garant [20] have suggested that the fibroblasts develop a tendency to fuse and form multinucleated cells in aged periodontal ligament. The possible histogenetic mechanism for giant fibroblast formation is summarized in Figure 4.

The presence of the giant fibroblasts clearly distinguishes the giant cell fibroma from an irritation fibroma and papilloma. The retrocuspid papilla may also contain giant cells similar to GCF, but is a site specific lesion. The important distinguishing features are mentioned in Table 1.

## Figures and Tables

**Table 1:** Illustrating comparison between giant cell fibroma, irritation fibroma, retrocuspid papilla, and papilloma.

	Giant cell fibroma	Irritation fibroma	Retrocuspid papilla	Papilloma
Etiology	Unknown	Chronic irritation	Developmental	Human papilloma virus
Age	1st–3rd decade	4th–6th decade	Children and young adult	30–50 years
Sex	Slight female predilection	Slight male predilection	Female predilection	Equal sex distribution
Common site	Gingiva, tongue	Buccal, labial, and tongue mucosa	lingual gingiva adjacent to mandibular cuspids. Frequently bilateral	Tongue, lips, and soft palate
Histopathology	Moderate to dense fibrous connective tissue stroma containing numerous giant cells, concentrated mostly beneath the epithelium; giant cells are stellate fibroblasts with enlarged nuclei and few containing multiple nuclei; surface epithelium typically has very elongated, thin rete processes	Dense, minimally cellular stroma of collagen fibers; stromal cells are bipolar fibroblasts with plump nuclei and fibrocytes with thin, elongated nuclei with minimal cytoplasm; surface epithelium is usually atrophic and may show signs of continued trauma	Connective tissue stroma may exhibit large stellate fibroblasts and occasional epithelial rests.	Keratinized stratified squamous epithelium arrayed in finger-like projections with thin fibrovascular connective tissue cores; koilocytes (virus altered epithelial cells) are sometimes seen high in the prickle cell layer
